# Subcutaneous phaeohypomycosis clinically presenting as bursitis

**DOI:** 10.3205/dgkh000639

**Published:** 2026-03-10

**Authors:** Anbuselvan Sivaranjani, Thanka Johnson, Ajitha Rajalingam, Natarajan Suresh

**Affiliations:** 1 Department of Pathology, Sree Balaji Medical College and Hospital, Chromepet, Chennai, Tamil Nadu India

**Keywords:** phaeohyphomycosis, subcutaneous abscess, bursitis

## Abstract

**Introduction:**

Phaeohyphomycosis is a spectrum of infections caused by dematiaceous pigmented fungi.

**Case report:**

A 57 year old man with uncontrolled type 2 diabetes mellitus presented with a well circumscribed swelling of the posterior aspect of the left elbow. Clinical work up was done and a provisional diagnosis of tuberculous bursitis was made. Excision of the lesion was done. Histopathological examination showed a fungal abscess with features of subcutaneous phaeohyphomycosis.

## Zusammenfassung

**Einleitung:**

Bei der Phäohyphomykose handelt es sich um ein Spektrum von Infektionen, die durch pigmentierte Schimmelpilze, auch Dematiaceae oder Schwärzepilze genannt, verursacht werden. Sie enthalten Melanin, das für die dunkle Verfärbung der Hyphen und Konidien sorgt.

**Fallbericht:**

Ein 57-jähriger Mann mit unkontrolliertem Typ-2-Diabetes mellitus stellte sich mit einer umschriebenen Schwellung des hinteren Teils des linken Ellenbogens vor. Nach der klinischen Untersuchung wurde die vorläufige Diagnose einer tuberkulösen Bursitis gestellt. Die Läsion wurde exzidiert. Die histopathologische Untersuchung ergab einen Pilzabszess mit Merkmalen der subkutanen Phäohyphomykose.

## Introduction

Phaeohypomycosis is a rare mycotic infection caused by various heterogenous groups of phaeoid (dermatiaceous) fungi involving the skin and subcutaneous tissue. Phaeohyphomycosis was first described in 1974 by Ajello et al. [1] to refer to superficial cutaneous infections, subcutaneous, disseminated, visceral, and systemic infections caused by slow-growing filamentous fungi with melanic pigment (phaeoid or dematiaceous) in their hyphae walls or spores. Common clinical manifestations are subcutaneous abscesses or cystic swellings [1], [2], [3]. We report a case of subcutaneous phaeohyphomycosis presenting as an asymptomatic cystic swelling over the bursa. Histopathology showed granulomatous inflammation and Grocott's methanamine silver stain revealed broad pigmented hyphae.

## Case report

**Figure 1:**
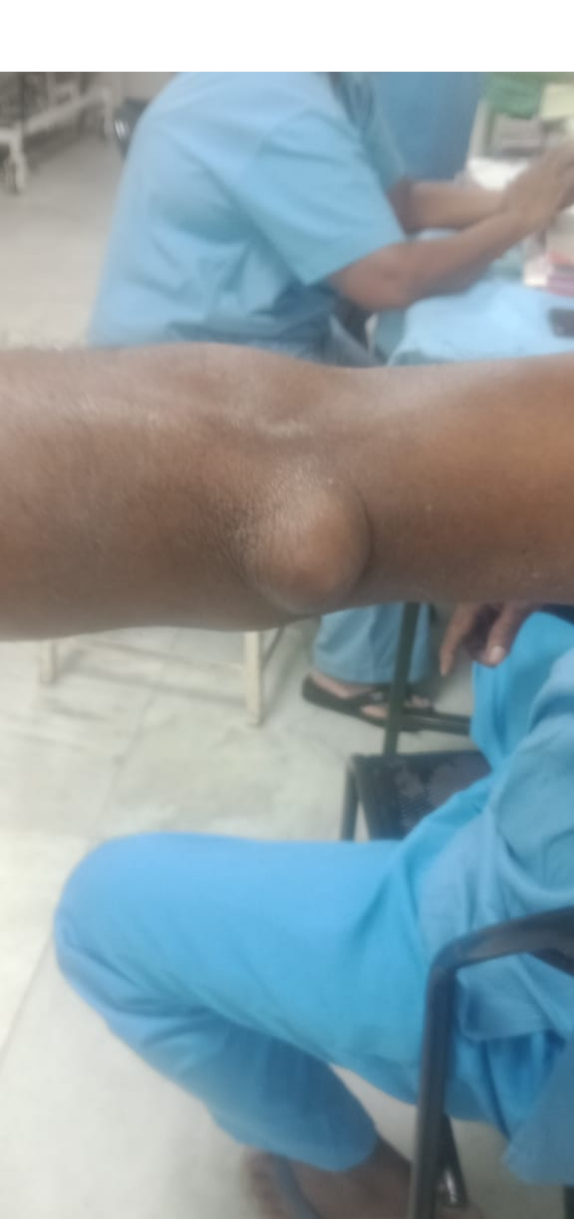
Clinical photograph of swelling over left elbow

A 57-year-old male complained of swelling on the posterior aspect of the left elbow for 10 years. Prior to that, the elbow was unremarkable. However, while playing a contact team sport known as Kabadi, he dove and fell on his left elbow, following which a swelling started appearing over his left elbow. The swelling’s onset was inconspicuous, but gradually progressed and attained a size of a lemon. The patient gave history of massaging the swelling himself; this temporarily reduced the swelling. It reappeared after 10 days and assumed the same size. There was no history of fever/weight loss/loss of appetite and no history of secondary skin change. The patient had type 2 diabetes mellitus, and was on medication. But this was ineffective. Local examination revealed a 6-cm x 6-cm spherical swelling over the posterior aspect of the elbow; it was mobile, cystic in consistency and non-reducable (Figure 1 (Fig. 1)). A clinical diagnosis of bursitis probably caused by tuberculosis was made.

**Figure 2:**
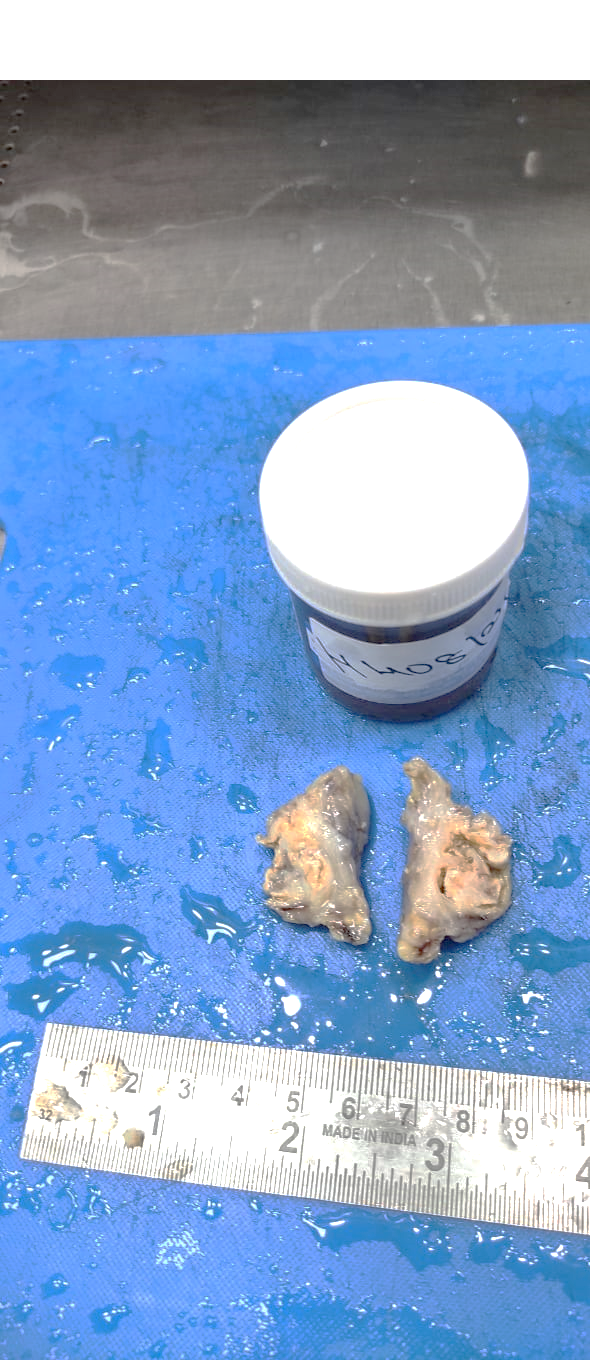
Excised swelling

Laboratory investigations showed uncontrolled diabetes with HbA1c was 11.67 despite the above-mentioned medication. An intradermal tuberculin test was done, which showed 13-mm induration. A cartridge-based nucleic acid amplification test (CB-NAAT/GeneXpert) was negative for tuberculosis. Fungal culturing was not performed, as clinically it was suspected as tuberculosis. Surgical excision was done. Gross tissue was grey-yellow, measuring 3 x 2 x 2 cm (Figure 2 (Fig. 2)).

**Figure 3:**
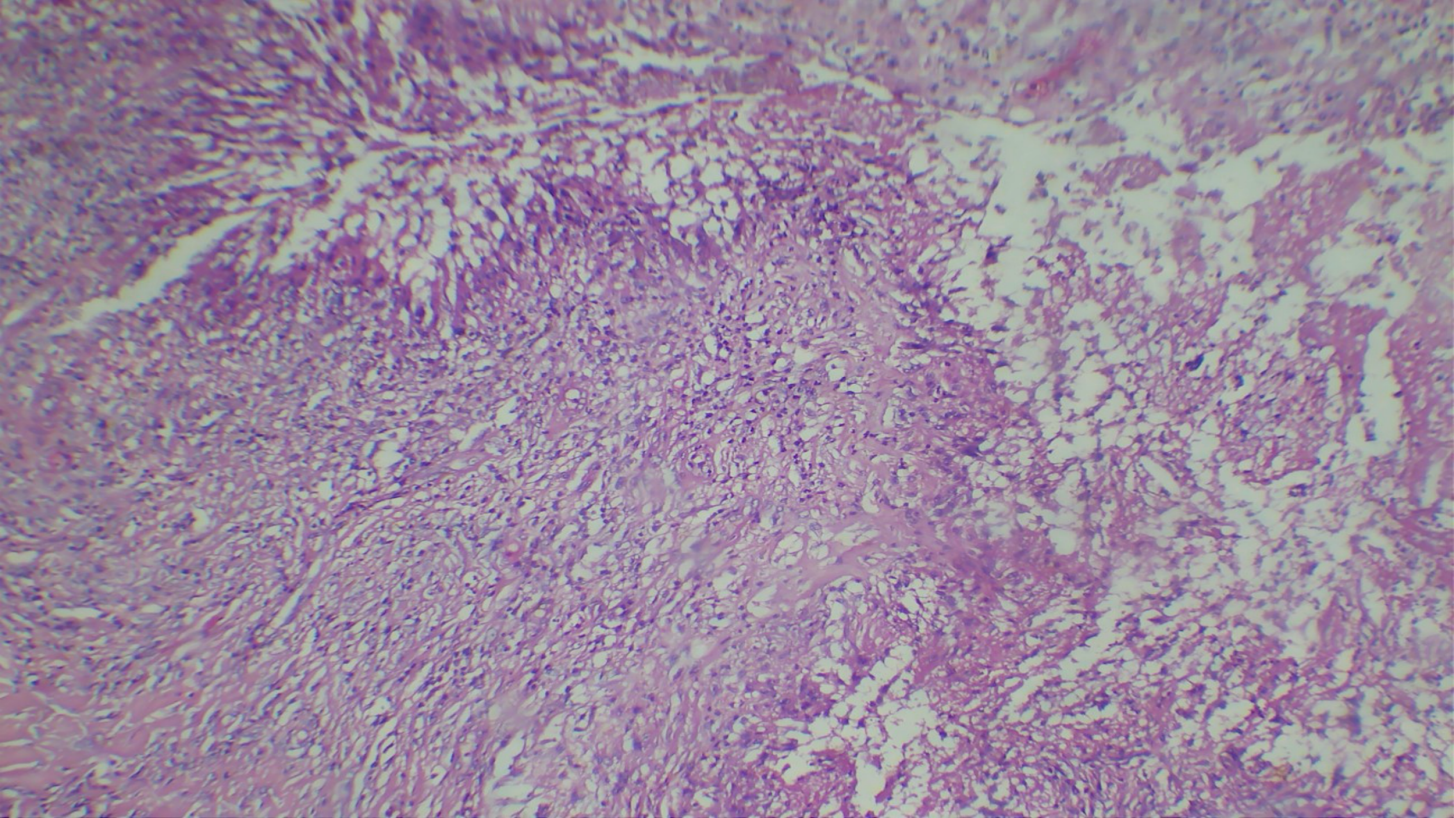
Hematoxylin and Eosin staining (40x) showing a necrotizing lesion with palisading granulomas containing giant cells

**Figure 4:**
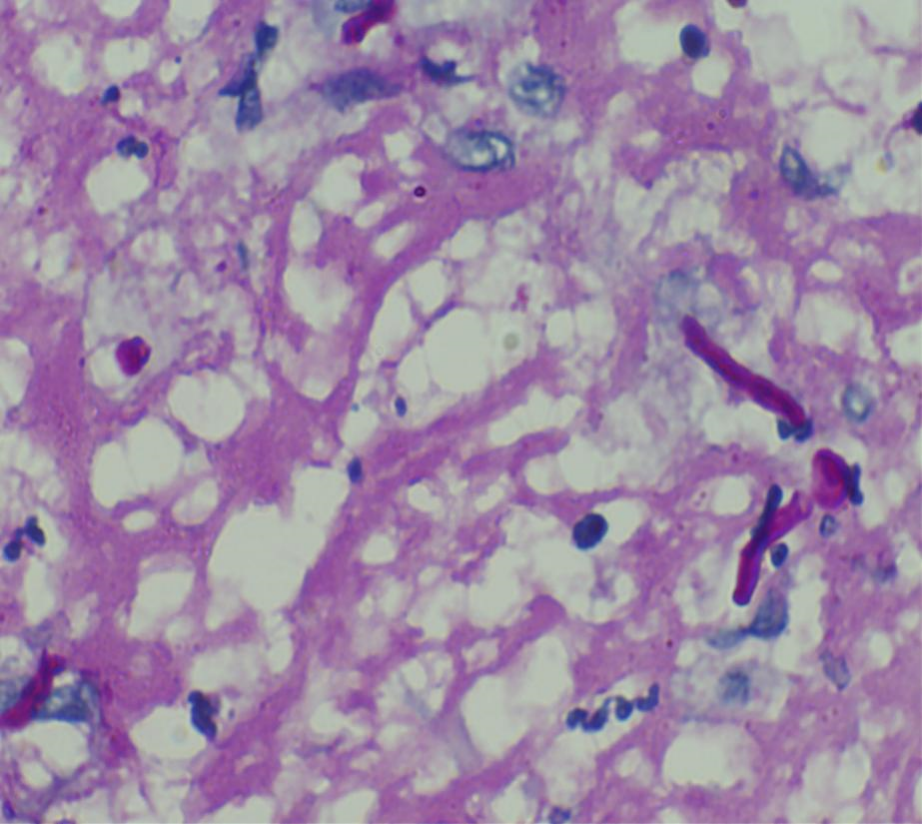
PAS-D staining (400x) showing branching, septate fungal hyphae

**Figure 5:**
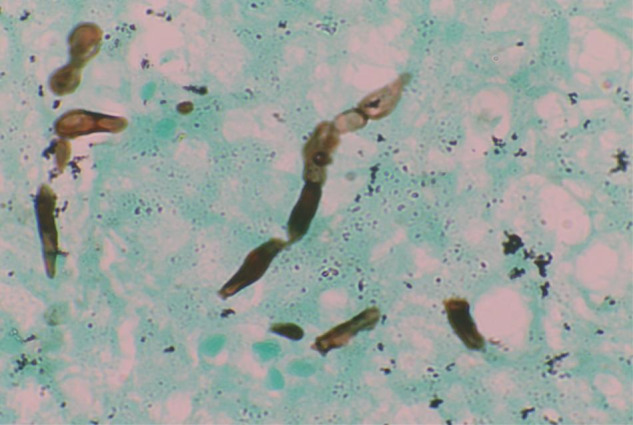
GMS staining (400x) showing branching, septate fungal hyphae with irregularly placed constrictions around septae

Upon microscopic examination, sections showed a well-circumscribed necrotizing lesion with palisading granulomas containing giant cells (Figure 3 (Fig. 3)). There were also areas of an old hemorrhage and cholesterol cleft formation. Staining for acid-fast bacteria was negative. The periodic-acid Schiff with diastase (PAS-D) stain (Figure 4 (Fig. 4)) and Grocott’s methenamine silver (GMS) stain both showed branching, septate fungal hyphae (Figures 5 (Fig. 5)). The diagnosis of a fungal abscess consistent with subcutaneous phaeohypomycosis was made, since the lesion was well circumscribed. No immediate antifungals started. Anti-diabetic measures were implemented and followed very strictly. The patient showed no recurrence or dissemination during 10 months of follow-up.

## Discussion

Phaeohyphomycosis represents a spectrum of infections caused by dematiaceous pigmented fungi. These infections are categorized into superficial, cutaneous, subcutaneous, systemic, and disseminated forms, with disseminated infections potentially affecting organs such as the brain, eyes, central nervous system, peritoneum, and bones. Subcutaneous phaeohyphomycosis primarily involves the limbs, fingers, wrists, knees, and ankles. Clinically, it may present as nodular or papular lesions, verrucous (wart-like) growths, hyperkeratotic plaques, ulcerated plaques, cysts, abscesses, pyogranulomatous lesions, chronic non-healing ulcers, or sinus tracts. The progression and severity of the disease depend on the host’s immune status, with immunocompetent individuals often showing localized lesions, while immunosuppressed patients are more likely to develop severe and disseminated infections [4], [5], [6].

Although subcutaneous phaeohyphomycosis is rare, its incidence is increasing, likely due to the growing number of immunocompromised patients. It usually develops following traumatic inoculation of contaminated material into the skin or subcutaneous tissue. Lesions are most frequently found on the hands and legs, particularly in outdoor workers. The infection primarily affects individuals aged 3 to 60, with males being more commonly affected due to occupational exposure. It is more prevalent in tropical and subtropical climates [1], [2].

The most common causative agents of subcutaneous phaeohyphomycosis are* Exophiala (E.) jeanselmei* and *E. dermatitidis*. These fungi, widely present in the environment, can infect immunocompromised individuals such as those with HIV, transplant recipients, or patients with chronic illnesses, diabetes, or those on immunosuppressive therapy. Immunocompetent individuals are less frequently affected. *E. jeanselmei* typically causes localized cutaneous or subcutaneous infections, often appearing as solitary lesions, such as phaeohypomycotic cysts. Even in severely immunosuppressed individuals. Exophiala infections tend to remain localized [7], [8], [9].

This case highlights the importance of considering fungal infections in the differential diagnosis of subcutaneous soft-tissue swellings, as these may be mistaken for conditions such as bursitis, lipoma, fibroma, epidermal cysts, or foreign-body reactions. Treatment usually involves surgical excision of the lesion, either alone or in combination with antifungal medications such as itraconazole, ketoconazole, or amphotericin B.

## Conclusion

Subcutaneous phaeohyphomycosis is characterized by the presence of branching, septate fungal elements in the subcutaneous tissue. Histopathological examination reveals granulomatous inflammation with septate hyphae. Diagnosis is confirmed by fungal stains and histological examination and special stains. This case was presented due to its rare clinical presentation as bursitis and to highlight the importance of histopathology.

## Notes

### Authors’ ORCIDs

- Sivaranjani A: https://orcid.org/0009-0000-2434-5060
- Johnson T: https://orcid.org/0000-0002-7499-5552
- Rajalingam A: https://orcid.org/0000-0002-5852-665X
- Suresh N: https://orcid.org/0000-0003-4966-5559

### Funding

None. 

### Competing interests

The authors declare that they have no competing interests.
